# Annotations

**Published:** 1908-12-12

**Authors:** 


### ANNOTATIONS.

### Remarkable Length of Days.

Our contemporary the *Medizinische Wochenschrift* of St. Petersburg gives some interesting particulars regarding a remarkable case of longevity. We need hardly say that our informant bears a reputation for the strictest integrity and the highest scientific accuracy. Nevertheless, we must disclaim any responsibility for the astonishing account which follows:—Andreas Schmidt was born on September 5, 1772, and served in the Reval Regiment for many years, taking part in the historic campaigns against Napoleon. In 1798 he accompanied Suworow's brigade across the Alps, and later on he took part in the skirmishes which the army of pursuit waged against the French troops retreating from Moscow. His military career is probably unique, as he remained on active service until he was eighty-six years of age, his final campaign being the Crimean War. In 1858 he was pensioned, and since then he has lived quietly, carrying the weight of his years well. He is able to go about, and talks and hears well. During the last few years, however, his sight has been gradually failing, and he has suffered from arthritic pains. To his medical interviewer he declared that he had never indulged in alcoholic drinks, and never smoked. His diet is by no means restricted, and even at present, when he is 136 years of age, he eats his meals with a hearty appetite.

### Bone-Setters' Advertisements.

Bone-setters succeed one another like the Amuraths. Some years ago a London surgeon gave an interesting account of one of the first of them, a personage of considerable notoriety in the 'seventies and 'eighties, who, he maintained, honestly believed himself a match for all the anatomists and pathologists in Europe. To judge by the advertisements now appearing in some of the halfpenny papers, self-confidence is still an attribute of these gentry. There is generally a three-quarter length photographic reproduction of a clean-shaven, frock-coated man of stern aspect, with letterpress made up chiefly of patients' testimonials. One is informed, however, that the only tools of this "bloodless surgeon" are his wondrous hands, "soft as velvet yet strong as steel." How these hands have learned their trade one may guess from the varied pursuits—often of a somewhat humble character—confessed to in a recent frank autobiography by such a kindred practitioner as "Dr." Walford Bodie of music-hall fame. And in appraising the value of the grateful letters help may perhaps be found in the unfortunate showing made lately by a bone-setter, in West End practice, in the correspondence columns of a medical contemporary. It finally appeared that one of his most successfully treated patients was still chronically disabled, and on that account in receipt of an allowance from the Army. In this particular case we agree to Mr. Bernard Shaw's proposition, that "the cure" is a persistent form of human delusion. The incident should not be without its lesson for those entering upon this branch of unqualified practice. For witness to the triumphs of his (almost literally) single-handed fight with disease, the bone-setter should rely upon those only who cannot be controverted—like, say, the faithful camel of Tartarin of Tarascon, which had seen its master kill all his lions.

### The Royal Society of Medicine.

The first Annual Dinner of the Royal Society of Medicine was held on the evening of December 4 at the Hôtel Cecil, and proved to be a great success. Sir William Church, the President of the Society, was in the chair, and there were present to support him distinguished representatives of many branches of public service, as well as a large number of prominent members of the medical profession. The toast of the Society was entrusted to Mr. Birrell, who, from his intimate personal knowledge of the difficulties that beset the amalgamation of different associations, was especially qualified to speak of the recent labours of the Royal Society of Medicine. As he very justly said, everyone nowadays desires to associate and to be bound together by bonds of union in federation. But the difficulty is to reconcile this desire with the general passion for autonomy: whilst everybody is willing to be associated with everybody else, he prefers to do so upon his own terms. Under these circumstances the results of the Society's efforts towards combination and co-ordination must be considered highly satisfactory. After only a few months its success may already be regarded as assured. During the past year of difficulty and trial there has been a large accession of Fellows and Members, and the Fellowship now numbers 2,215 and the Membership 959. The library of the Society, whose nucleus may be said to be the valuable collection of the Royal Medical and Chirurgical Society, now contains 100,000 volumes, and forms one of the most important medical, scientific and historical collections in the world. The President in his reply, laid particular emphasis on the fact that the Society is not the Royal Society of Medicine of London, but of the United Kingdom and the British Empire. Of the 3,174 Fellows and Members, about 1,780 live in London, 1,170 are in the provinces, and 220 are scattered over the face of the globe. The amalgamation has already met with much success, but to make it truly representative of British Medicine and of the sciences upon which medicine is based, a great deal of effective work must be gone through. Various small societies, to whose advantage it would surely be to join forces with the larger organisation, still remain outside the fold. Their reasons for retaining complete independence are easy to understand, and must command our sympathy; but, now that judicious centralisation has proved itself and shown that its intentions are anything but oppressive, the principal objection to amalgamation is removed. The case of the Medical Society of London is on a different footing, and it is probably well advised to continue its separate existence.

